# Musculoskeletal Coccidioidomycosis in the Setting of Adalimumab: A Case Report

**DOI:** 10.7759/cureus.56321

**Published:** 2024-03-17

**Authors:** Ashkon Nehzati, Donald Hefelfinger, Elizabeth Fonte, Joshua Scott

**Affiliations:** 1 Internal Medicine, Boonshoft School of Medicine, Wright State University, Dayton, USA; 2 College of Medicine, Boonshoft School of Medicine, Wright State University, Dayton, USA; 3 Rheumatology, Wright-Patterson Medical Center, Dayton, USA

**Keywords:** adalimumab, valley fever, coccidioides immitis, disseminated coccidioidomycosis, musculoskeletal coccidioidomycosis

## Abstract

Musculoskeletal coccidioidomycosis is a rare disseminated fungal infection caused by either *Coccidioides immitis* or *Coccidioides posadasii *endemic to the southwestern United States and northwestern Mexico, as well as Guatemala, Brazil, and other locations in Central and South America. Symptomatic primary infection of coccidioidomycosis can present as pneumonia with influenza-like symptoms, but the majority of cases remain asymptomatic. When dissemination occurs, the most common extrapulmonary sites include the skin, lymph nodes, musculoskeletal system, and meninges. We present a case of a 53-year-old female with a history of breast cancer and ankylosing spondylitis treated with adalimumab who presented with disseminated coccidioidomycosis. On presentation, she reported subcutaneous nodules on the right forearm and elbow. Radiologic evaluation utilizing magnetic resonance imaging (MRI) and positron emission tomography (PET) scan revealed multiple subcutaneous and bony enhancing lesions in her right forearm, lumbar spine, iliac wing, and axillary lymphadenopathy. Given the patient's history of breast cancer, there was concern for metastatic disease. Axillary lymph node biopsies were negative for malignancy, but immunoreactive for *C. immitis* with a positive Grocott methenamine silver (GMS) stain and a *C. immitis* antibody panel confirmed the diagnosis of disseminated coccidioidomycosis. Treatment with fluconazole was initiated along with discontinuation of adalimumab. Fluconazole was transitioned to itraconazole due to adverse effects. Treatment was successful as evidenced by improved PET imaging and downtrending *C. immitis* antibody titers. This case highlights the concerning potential for dissemination of endemic mycoses with anti-tumor necrosis factor-α (TNF-α) therapies and the unique ways in which they can present. Further investigation is needed to determine the long-term implications of the disease and the role that immunosuppressive medications play in disease susceptibility.

## Introduction

Coccidioidomycosis, also known as San Joaquin Valley Fever, is a fungal infection that results from inhalation of aerosolized spores produced by either *Coccidioides immitis* or *Coccidioides posadasii* [1-10]. These dimorphic fungi are endemic in the southwestern United States and northwestern Mexico, as well as Guatemala, Brazil, and other locations in Central and South America where they typically reside in the soil [1-10]. The primary infection typically presents as community-acquired pneumonia with influenza-like symptoms, but less than 40% of patients become symptomatic [2,3,6,7,9,11]. Pulmonary complications occur in approximately 5-10% of cases and typically present as persistent pulmonary nodules or cavitary lesions [1-3,6,7,9]. Common risk factors for developing symptomatic coccidioidomycosis are male gender, pregnancy, immunosuppression, and African or Pacific Islander ancestry [1,2,5,6,9,10]. Dissemination of coccidioidomycosis is exceedingly rare and occurs in less than 1% of patients by way of hematogenous or lymphatic spread [1-4,6]. The most common extrapulmonary sites of dissemination include the skin, lymph nodes, musculoskeletal system, and meninges [1-4,6,8]. Approximately 20-50% of disseminated infections involve the musculoskeletal system [1-4,6,8].

When musculoskeletal coccidioidomycosis is present, the knee is most commonly involved, but any bone or joint may be affected [1-4,6,8]. The clinical findings in patients with musculoskeletal coccidioidomycosis are typically nonspecific and include localized pain, arthralgias, swelling, or warmth of the affected bone or joint [1-4,6,8]. Thus, early diagnosis and management of this condition require a high index of suspicion by the medical team to help minimize unnecessary interventions. We present a rare case of a 53-year-old female who was diagnosed with disseminated coccidioidomycosis in the setting of immunosuppressive treatment for ankylosing spondylitis. This case outlines the difficulties that arise in the diagnosis and treatment of musculoskeletal coccidioidomycosis and illustrates the challenge of deciding the most appropriate rheumatologic therapy after establishing the diagnosis.

## Case presentation

The patient is a 53-year-old female with a past medical history of ankylosing spondylitis treated with adalimumab and a remote history of stage 1 estrogen receptor (ER)/progesterone receptor (PR) negative, human epidermal growth factor receptor 2 (HER2)-neu positive, grade 3 breast cancer in 2011 treated with lumpectomy and adjuvant brachytherapy currently in remission. Tumor necrosis factor-α (TNF-α) inhibition was selected by her previous treating rheumatologist for the iritis component of ankylosing spondylitis and was continued based on good control of enthesitis, inflammatory back pain (low bath ankylosing spondylitis disease activity index (BASDAI) of 2.7), and no recurrence of iritis. She presented in follow-up to the rheumatology clinic with a four-month history of palpable subcutaneous nodules in her right forearm and elbow. There was radiographic evidence of lytic lesions in the lumbar spine, right iliac wing, and axillary lymphadenopathy. Of note, she is a bioenvironmental engineer with travel to locations in the United States including Arizona and various international locations where she frequently worked in burn pits. 

**Figure 1:**
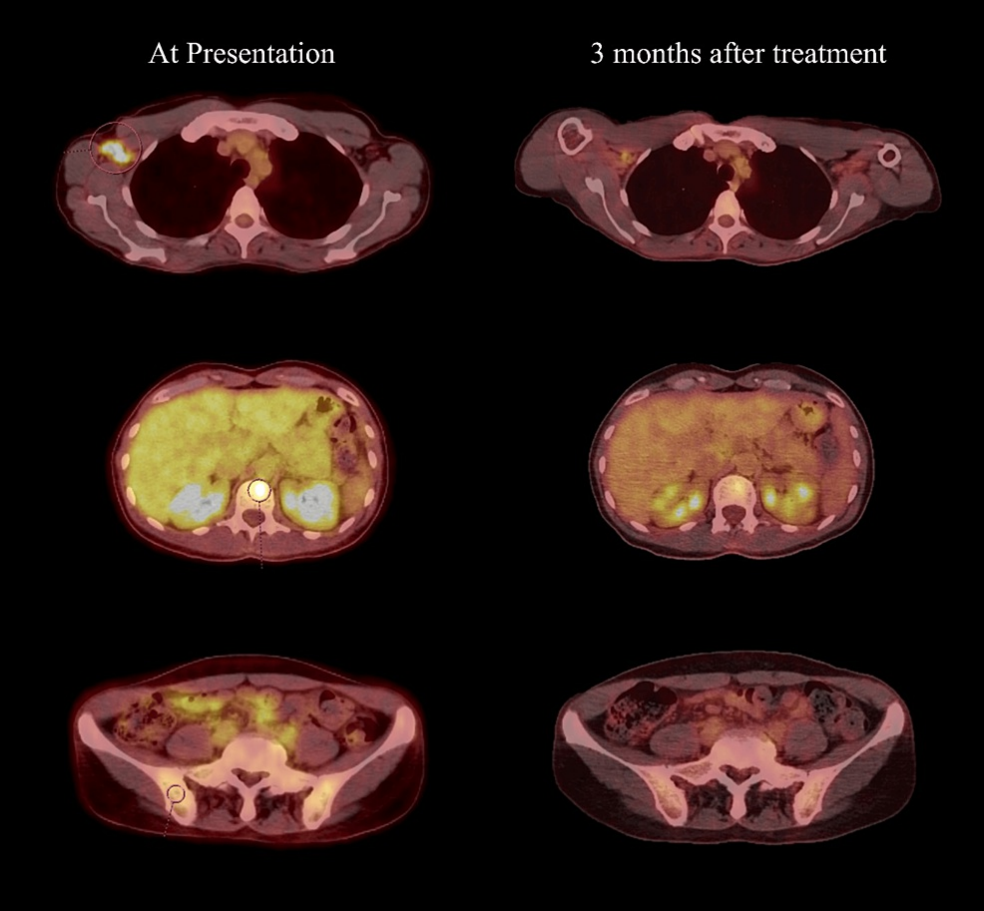
PET scan of patient PET scan at presentation (left column) showing FDG avidity in the left axilla (top), axial skeleton (middle), and right iliac wing (bottom) with a significant decrease in all sites after treatment initiation (right column). Areas of avidity noted by circles in each image at presentation. PET, positron emission tomography; FDG, fluorodeoxyglucose

The patient initially presented to her oncologist with complaints of subcutaneous right forearm and elbow nodules that had not responded to treatment with cephalexin and clindamycin at an urgent care visit. At that time, she was noted to have a rash with pruritus on her right forearm that symptomatically improved temporarily with over-the-counter medications. Without symptomatic improvement after empiric antibiotics and elevated c-reactive protein of 1.25 mg/dL, a positron emission tomography (PET) scan and magnetic resonance imaging (MRI) of the right arm and lumbar spine were ordered. MRI of the right arm showed multiple small well-circumscribed soft tissue lesions concerning metastatic disease. MRI of the lumbar spine and pelvis showed hypointense enhancing lesions of the lumbar spine (L1 vertebral body, L4-5, and L5-S1 endplates) as well as right iliac wing lesions. Due to concern of recurrent malignancy, a PET scan was ordered demonstrating right axillary conglomeration of abnormal lymph nodes and small abnormal foci in the right iliac wing and L1 vertebral body concerning metastatic disease (Figure 1). With multi-focal PET avid lesions, excisional biopsy of the axillary lymph node demonstrated necrotizing granulomas with negative Grocott methenamine silver (GMS)/Acid-fast bacilli (AFB) stains. Further biopsy of the L1 vertebral body was recommended to establish a definitive diagnosis. 

A broad differential included autoimmune disease, malignancy, and atypical infections such as *Coxiella*, *Mycobacteria*, blastomycosis, histoplasmosis, or coccidioidomycosis. After consultation with infectious disease, existing lymph node samples were sent out for mycobacterial and fungal testing. Serologic testing was performed for *Histoplasma*, *Blastomyces,* and *Coccidioides* with a plan for biopsy of the L1 vertebra for additional cultures if needed. A complete blood count with differential was unremarkable. Further testing of the lymph node samples was immunoreactive for *C. immitis* with a positive GMS stain. *C. immitis* antibody panel confirmed the diagnosis of disseminated coccidioidomycosis with multiple lesions involving the subcutaneous right forearm, elbow, lumbar spine, axillary lymph nodes, and right iliac wing. 

Adalimumab was discontinued on confirmation of *Coccidioides* infection and treatment with fluconazole was promptly started. Fluconazole was transitioned to itraconazole due to adverse effects of hair loss and phantogeusia. The patient continued treatment with itraconazole demonstrating downtrending *C. immitis* antibody titers from 1:32 to <1:2 over the next 18 months. Treatment of the patient’s coexisting ankylosing spondylitis with celecoxib, physical therapy, and lifestyle modifications has maintained disease control with a BASDAI of 2.0. In addition, there has been no radiographic progression of ankylosing spondylitis and only rare mild iritis managed by ophthalmology with topical corticosteroids.

## Discussion

*Coccidioides* is a highly pathogenic dimorphic fungal species endemic to the southwestern United States [8,9]. It is thermally dimorphic, presenting as a mold in cold soil, and is transformed into a yeast inside of its host due to higher temperatures within the body [10]. The mold releases arthroconidia when soil is disturbed, and infection is achieved through inhalation of the aerosolized arthroconidia [10]. In vivo, the arthroconidia mature into spherules that contain hundreds of endospores that result in subacute or acute pneumonia [10]. The exact mechanism of endospore and spherule destruction is unknown but immune response relies on both innate and adaptive immunity with an interplay between CD4+, CD8+, TH1, TH2, and TH17 cells [8,10]. Immunocompromised individuals are at an increased risk of dissemination due to low CD4+ cell counts, but a decrease in total IL-10 has a protective role in resistance to infection [8,10]. *C. immitis’* presentation is similar to that of many other infections and thus high clinical suspicion in susceptible populations (elderly, immunocompromised, travelers to endemic areas) is essential to early diagnosis and prevention of dissemination and long-term complications [8,9]. Musculoskeletal lesions only account for a small portion of disseminated cases but their unique pathology poses a challenge for clinical diagnosis and treatment as the differential is vast and the treatment guidelines are unclear.

TNF-α inhibitors in particular are associated with increased risk of opportunistic infections. TNF-α is essential for the formation and maintenance of granulomas [12]. It primes macrophages for intracellular killing, along with other cytokines and chemokines, and it induces the recruitment and organization of mononuclear cells into mature granulomas [12]. TNF-α blockade leads to a failure of containment of intracellular pathogens, which predisposes the host to opportunistic granulomatous infections [12].

The differential diagnosis for musculoskeletal coccidioidomycosis is broad and requires a high index of suspicion based upon factors such as immunocompromised status, travels to endemic regions, and presenting symptoms as it tends to mimic other osseous pathologies [1,6]. This differential includes multiple myeloma, metastatic osteolytic disease, tuberculosis, Kaposi sarcoma, atypical mycobacterial infections, and various other disseminated fungal infections [2,6]. The initial diagnostic evaluation for musculoskeletal coccidioidomycosis involves the use of plain radiographs, which demonstrate well-circumscribed lytic lesions [2,6]. However, CT and MRI are the preferred imaging modalities for detecting skeletal and soft tissue abnormalities and determining the best site for biopsy [2,6,7]. Serologic studies are also useful in establishing a diagnosis by detecting anti-coccidioidal antibodies as they have a high sensitivity and specificity [1]. Differentiating between *C. immitis* and *C. posadasii* is so far only possible with genetic testing [1]. Definitive diagnosis can be established with a biopsy of the skeletal or soft tissue lesion. Histologic examination of the above-mentioned biopsy sample will demonstrate characteristic spherules that contain hundreds of endospores [4,6,7]. These endospores can initiate a neutrophilic inflammatory reaction followed by a non-caseating granulomatous response with giant cells that closely resemble tuberculosis [6]. Due to the vast similarities that musculoskeletal coccidioidomycosis has with other skeletal pathologies, establishing a diagnosis early in its development is often difficult, and improper treatment modalities are frequently used. 

The current treatment guidelines for musculoskeletal coccidioidomycosis do not make clear recommendations regarding the management of non-vertebral bone and joint coccidioidal infections [11]. Experts state that proper treatment ultimately depends on the severity of the disease process and the need for surgical debridement [9]. Cases of disseminated coccidioidomycosis are generally treated with a course of azole antifungal therapy, with a preference for itraconazole, for at least six months, but many cases will require prolonged to lifelong antifungal therapy as well as the possible need for surgical debridement [5,7,9]. Amphotericin B is also an effective treatment option, especially for patients who have critical lesions, are rapidly worsening, or do not respond to initial azole therapy, but this medication carries a wide range of adverse effects [5,6,9]. It has been shown that the combination of antifungal therapy and surgical intervention leads to remission in a large portion of cases, but more than one procedure is often required which can result in abnormalities of surrounding structures [7]. There is also debate regarding the utilization of multidrug therapy for the treatment of severe coccidioidomycosis, but its use remains controversial due to the increased risk of drug-drug interactions [2,9]. While the mortality for patients with musculoskeletal coccidioidomycosis is low, morbidity for these patients is high due to the possible need for multiple surgical debridement procedures with a high incidence of recurrence [6,7]. Due to a lack of clear treatment guidelines for musculoskeletal coccidioidomycosis, there is a significant barrier in the management of this disease and prevention of recurrence in the future. 

While our patient has maintained remission of axial and ophthalmologic disease manifestations on celecoxib alone, thoughtful consideration of the next steps for treatment is needed. If rheumatologic disease progression occurs, other treatment modalities need to be investigated in the setting of simultaneous coccidioidomycosis. Current literature recommendations are based on published case studies and there is a need for continued research to help determine evidence-based guidelines. Upon literature review, retrospective case studies have demonstrated after appropriate treatment with anti-fungal therapy, resumption of immunosuppressive medication by day 30 of treatment, did not result in dissemination or coccidioidomycosis disease progression [13]. This suggests that coccidiomycosis can be treated despite the use of biologics or immunosuppressive medications. Other recommendations include continuing or restarting biologics if the patient remains asymptomatic with simultaneous anti-fungal therapy for at least six to 12 months and repeated negative serology for coccidioidomycosis in one to three months or positive with demonstrated inactive disease [14]. Another factor that may increase the risk of severe coccidioidomycosis is the use of long-term corticosteroids with its related transient state of immunosuppression and ability to help lessen immune exacerbations of rheumatic diseases [14].

## Conclusions

Musculoskeletal coccidioidomycosis is a disease that closely resembles a multitude of other pathologies and, therefore, requires a high clinical suspicion to establish an early diagnosis to prevent complications of advanced disease. Limited treatment guidelines exist for the management of disseminated coccidioidomycosis with musculoskeletal involvement, so close surveillance is needed to ensure disease resolution and guide changes in management. This case is further complicated by concurrent ankylosing spondylitis and despite successful treatment of disseminated coccidioidomycosis, there are long-term challenges in the management of rheumatic disease activity. Further investigation is needed to determine and characterize the long-term implications of this disease and the role immunosuppressive medications can play in susceptibility. This case highlights the concerning potential for dissemination of endemic mycoses with anti-TNF-α therapies and the unique ways in which they can present.
